# Left ventricular noncompaction in Duchenne muscular dystrophy

**DOI:** 10.1186/1532-429X-15-67

**Published:** 2013-08-01

**Authors:** Christopher J Statile, Michael D Taylor, Wojciech Mazur, Linda H Cripe, Eileen King, Jesse Pratt, D Woodrow Benson, Kan N Hor

**Affiliations:** 1 Cincinnati Children’s Hospital Medical Center, Cincinnati, OH, USA; 2 The Ohio Heart and Vascular Center at The Christ Hospital, Cincinnati, OH, USA; 3 Nationwide Children’s Hospital, Columbus, OH, USA; 4 Children’s Hospital of Wisconsin, Milwaukee, WI, USA

**Keywords:** Left ventricular noncompaction, Duchenne muscular dystrophy, Cardiac magnetic resonance imaging

## Abstract

**Background:**

Left ventricular noncompaction (LVNC) describes deep trabeculations in the left ventricular (LV) endocardium and a thinned epicardium. LVNC is seen both as a primary cardiomyopathy and as a secondary finding in other syndromes affecting the myocardium such as neuromuscular disorders. The objective of this study is to define the prevalence of LVNC in the Duchenne Muscular Dystrophy (DMD) population and characterize its relationship to global LV function.

**Methods:**

Cardiac magnetic resonance (CMR) was used to assess ventricular morphology and function in 151 subjects: DMD with ejection fraction (EF) > 55% (n = 66), DMD with EF < 55% (n = 30), primary LVNC (n = 15) and normal controls (n = 40). The non-compacted to compacted (NC/C) ratio was measured in each of the 16 standard myocardial segments. LVNC was defined as a diastolic NC/C ratio > 2.3 for any segment.

**Results:**

LVNC criteria were met by 27/96 DMD patients (prevalence of 28%): 11 had an EF > 55% (prevalence of 16.7%), and 16 had an EF < 55% (prevalence of 53.3%). The median maximum NC/C ratio was 1.8 for DMD with EF > 55%, 2.46 for DMD with EF < 55%, 1.54 for the normal subjects, and 3.69 for primary LVNC patients. Longitudinal data for 78 of the DMD boys demonstrated a mean rate of change in NC/C ratio per year of +0.36.

**Conclusion:**

The high prevalence of LVNC in DMD is associated with decreased LV systolic function that develops over time and may represent muscular degeneration versus compensatory remodeling.

## Background

Left ventricular noncompaction (LVNC) is characterized by deep trabeculations in the left ventricular (LV) endocardium. The LVNC phenotype has been described in several genetically mediated diseases including the alpha-dystrobrevin mutation, mitochondrial mutations, and Cypher/ZASP mutation [1,2]. In this setting, LVNC has been thought to be a primary process resulting from arrest in normal myocardial compaction during human embryonic development between weeks 5–8 [3]. LVNC has been associated with other cardiac phenotypes, including LV hypertrophy, LV dilation and combined LV hypertrophy and dilation. LVNC has been described in genetic disorders with cardiac involvement, e.g. G4.5 (Barth syndrome) and Duchenne Muscular Dystrophy (DMD) [4-6]. The extent to which LVNC is a primary defect or is a secondary, compensatory process in these genetic disorders remains unclear [7,8].

Patients with DMD, a lethal X-linked skeletal and cardiac myopathy caused by dystrophin mutations, universally develop cardiac dysfunction [9]. Echocardiography is used to assess function in younger DMD patients; however, due to poor windows, cardiac magnetic resonance imaging (CMR) is used to monitor function in older patients. CMR is also a sensitive tool for the diagnosis of LVNC [10-16]. In the course of performing several hundred DMD cardiac MR studies, a marked prevalence of LVNC was noted. This study aimed to define the prevalence of LVNC in DMD patients and characterize its relationship to global LV function using CMR. LVNC imaging criteria were used to evaluate the extent of LVNC in these studies. Specific CMR criteria for LVNC have been proposed, but there are no universally accepted guidelines. A common criteria uses an anatomic measurement of non-compacted (NC) to compacted (C) (NC/C) ratio > 2.3:1 in diastole as the threshold to distinguish pathologic LVNC [14].

## Methods

### Study population

Data was analyzed from records of patients with DMD or LVNC followed at Cincinnati Children’s Hospital Medical Center (IRB). The diagnosis of DMD was confirmed by skeletal muscle biopsy in all patients. The diagnosis of LVNC was made clinically based on accepted non-invasive imaging criteria. All DMD or LVNC patients that underwent CMR between 2005 and 2010 were included. An age-matched control group of subjects with completely normal CMR finding was also analyzed. For patients with more than one CMR study, the most recent study was used for the primary analysis. Serial data was evaluated for all DMD patients with multiple CMR studies. The study was approved by our Institutional Review Board.

### Imaging protocols

All CMR studies were performed according to standard clinical protocol. Studies included standard cardiac functional imaging using a retrospective vectorcardiographic (VCG)-gated segmented steady-state free precession (SSFP) sequence. Patients were imaged on both a Siemens Avanto 3 T and GE 1.5 T magnets. A 3 T standard imaging was preceded by frequency scouting and adjustment. Subjects were breath-held as tolerated. For those subjects who could not adequately breath-hold, a free breathing technique with multiple signal averages was used. Standard functional imaging included a short-axis stack of cine SSFP images from cardiac base to apex. The short axis was prescribed as the perpendicular plane to the LV long axis in 2- and 4-chamber views as previously described [17,18]. Typical scan parameters included field of view 32 to 38 cm, slice thickness 5 mm, echo time/repetition time(TE/TR) 1.4/2.8 (Siemens Medical Solutions), TE/TR 2.0/4.0 (GE Healthcare), and in-plane resolution was 1.5 mm. A minimum of 12 slices were performed, with 24 phases/slice. The typical temporal resolution of the cine SSFP images was 30 to 40 ms, and was adjusted according to the patient’s heart rate and ability to breath-hold. Myocardial Delayed Enhancement (MDE) Imaging was performed when intravenous (IV) access was obtained; at our center, IV access is routinely obtained in all DMD subjects if possible, though in DMD subjects < 10 years of age, IV gadolinium is deferred if successful IV placement requires more than 1 venipuncture attempt. MDE imaging was performed via a gradient echo inversion sequence recovery protocol 10 min after 0.2 mmol/kg gadolinium diethylenetriamine penta-acetic acid (Gd-DTPA) injection. MDE imaging was considered positive if any area of the myocardium showed hyperenhancement as assessed by consensus of 2 independent expert observers.

### Global functional data

Ventricular volumes, mass, and global function were assessed via standard planimetry with a semi-automated technique (QMASS version 6.1.5, Medis Medical Imaging Systems, Leiden, the Netherlands) [19,20]. This assessment was performed on images from either scanner, independent of vendor or field strength [21]. Late gadolinium enhancement (LGE) status for each segment, ventricular volumes, mass, and EF were tabulated for each subject and then exported to a RedCap database.

### LVNC assessment

**Figure 1:**
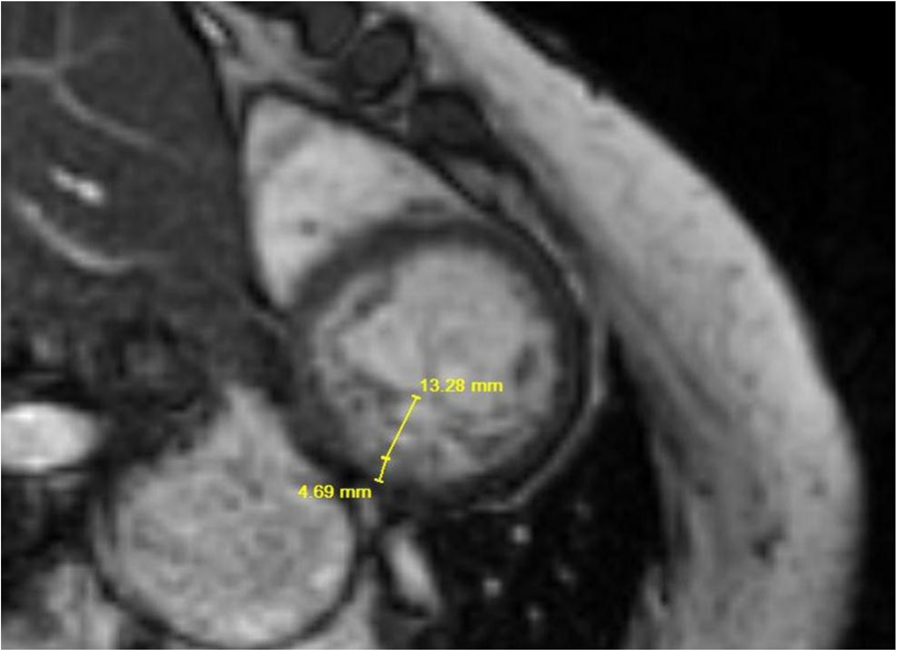
Example of measurement of noncompacted: compacted ratio in the short axis view of CMR of a DMD patient.

The LVNC status was assessed according to a standard metric previously described [14]. Using a high fidelity digital ruler within AMICAS PACS, the non-compacted to compacted ratio (NC/C) was measured in all 16 segments according to the AHA 16 segment model. The apical cap was not included in the assessment. All measurements were made by the primary investigator. The myocardium was considered to be positive for LVNC if the noncompacted to compacted ratio was greater than 2.3:1 in diastole in one segment as described by Petersen et al. [14]. A similar technique was used by Dawson et al. in a study describing the characteristics of compacted and non-compacted ratios [22] (Figure 1). The maximal NC/C ratio was defined as the largest NC/C in any segment. Care was taken to avoid areas of multihead attachment of the papillary muscles to the left ventricle [23].

### Pathologic correlation

Three of the patients enrolled in the study (1 DMD and 2 primary LVNC) had specimens available for image correlation. The DMD patient underwent placement of a HeartMate left ventricular assist device and both primary LVNC patients underwent orthotopic heart transplantation. An experienced pathologist analyzed the specimens for the presence and extent of LVNC.

### Statistical analysis

Prevalence of LVNC was compared using a Chi-Square test and prevalence for each group was estimated using a 95% Wald confidence interval for proportions. Spearman’s Rank correlation was used to determine association between the left ventricular end diastolic volume Z-score and NC/C ratio. Median ratios were compared using the Wilcoxon Rank-Sum test when two groups were present and the Kruskal-Wallis test for more than two. Inter- and intra- observer agreement was measured using the intraclass correlation coefficient (ICC). These values range from 0 to 1, with 0.0-0.2 indicating very little agreement, 0.2-0.4 little agreement, 0.4-0.6 moderate agreement, 0.6-0.8 strong agreement, and 0.8-1.0 very strong agreement. For all tests, a p-value of < 0.05 was considered statistically significant. Corrections for multiple comparisons were made using the Bonferroni method. All analyses were performed using SAS version 9.2 (SAS Institute Inc., Cary, NC).

## Results

### Study population

There were a total of 151 subjects enrolled; DMD (n = 96), normal control (n = 40) and primary LVNC (n = 15). A total of 229 CMR studies were evaluated including 78 serial DMD studies. There were 96 DMD boys: EF > 55% (n = 66) and EF < 55% (n = 30). All 40 normal control subjects had an EF > 55%. Of the 15 patients with primary LVNC, 6 had an EF < 55% and 9 with an EF > 55%. The median ages of the groups are included in Table 1.

**Table 1:** Demographic data of the study population

**Group**	**Total number**	**Median age**	**# Male (%)**
DMD with EF >55%	66	13.7	66 (100)
DMD with EF <55%	30	15.1	30 (100)
Primary LVNC	15	17.6	8 (53.3)
Normal	40	16	22 (55)

### LVNC in DMD

Among the 96 DMD boys, there were 27 cases meeting the LVNC criterion, with an overall prevalence of 28% (95% CI = 19.1% - 37.1%, p < 0.0001). Of the 27 LVNC cases in DMD boys, 11 had an EF > 55%, and 16 with an EF < 55%. The prevalence of LVNC in DMD boys with EF > 55% was 16.7% (95% = 6.8% - 26.6%) compared to 53.3% (95% = 35.4% - 71.2%) for DMD boys with EF < 55% (p = 0.0002). No normal subject had evidence of LVNV or an NC/C ratio > 2.3 in any segment.

**Figure 2:**
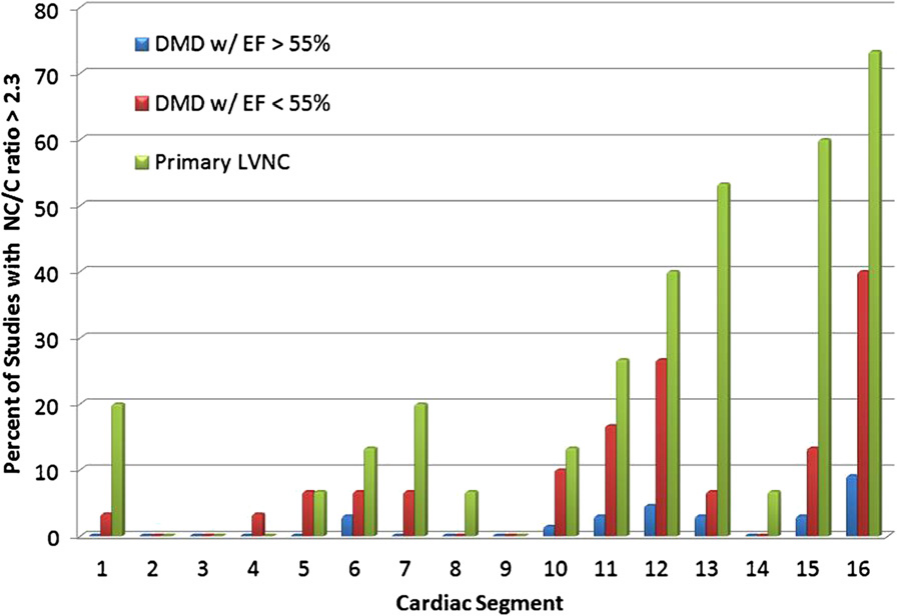
**Distribution of positive segments by patient group.** Segment 16 (apical lateral) in both DMD and LVNC most commonly fulfilled criteria for LVNC. The apical and lateral segments are more frequently positive across all groups which is consistent with previous findings in LVNC. DMD, Duchenne Muscular Dystrophy; EF, ejection fraction; LVNC, left ventricular non-compaction; NC, noncompacted length; C, compacted length.

Particular LV segments that were positive for LVNC with a NC/C ratio > 2.3 were in similar locations in patients with DMD with EF > 55%, DMD with EF < 55% or LVNC. In all the subjects, the apical segments were more likely to have ratios > 2.3 with segment 16 being the most common segment to be positive. Segment 16 was positive in 73% of the primary LVNC patients, 40% of DMD subjects with an EF < 55%, and 9.1% of DMD subjects with an EF > 55%. The segments most commonly positive in the LVNC population were apical segments 13, 15 and 16. In the DMD population, it was segments 11,12 and 16. These segments correspond to the segments that have been described to have scar tissue burden with LGE analysis [24] (Figure 2).

**Figure 3:**
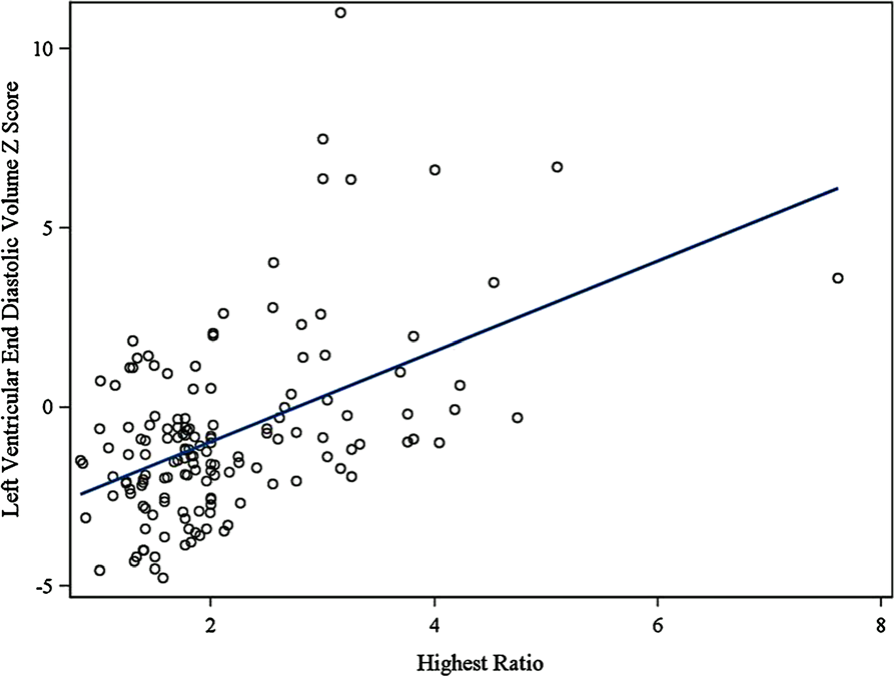
**LVED Z-score vs NC/C ratio.** There is a linear relationship with r = 0.40 (p < 0.0001). As the LVED increases the NC/C ratio also increases. DMD, Duchenne Muscular Dystrophy; LVEDV, left ventricular end diastolic volume; NC, noncompacted length; C, compacted length.

Consistent with the previous data which suggests that there is a higher prevalence of LVNC in the DMD boys with decreased function, there was a correlation between left ventricular end diastolic volume Z- score and NC/C ratio (Figure 3). The relationship was linear with an r = 0.40 (p <0.0001). As the left ventricular end diastolic volume z-score increased so did the NC/C ratio.

**Figure 4:**
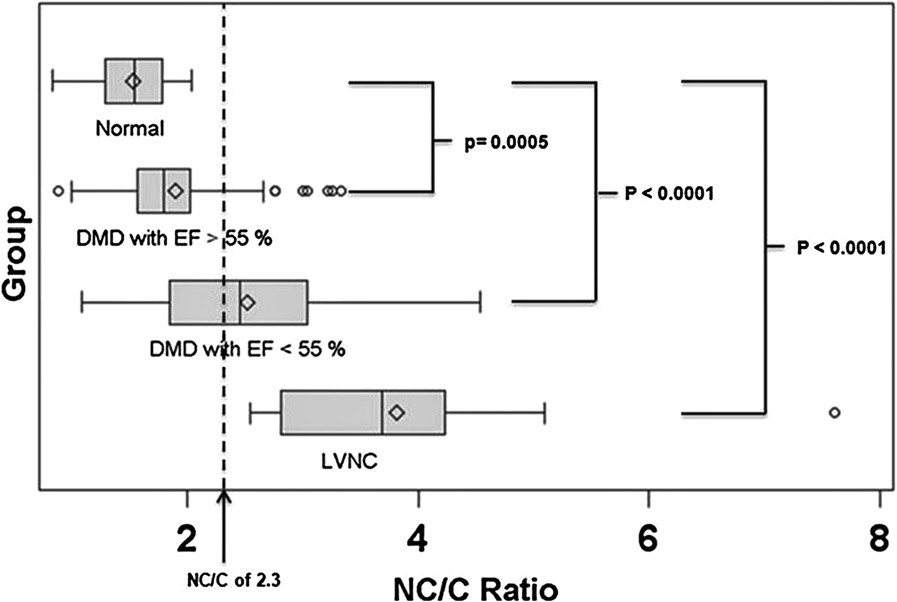
**Noncompacted to compacted ratio.** This is a box plot showing the mean (diamond), median (middle vertical line), as well as interquartile range (end of the boxes) of the highest NC/C ratio for each population. DMD, Duchenne Muscular Dystrophy; EF, ejection fraction; LVNC, left ventricular noncompaction; NC, noncompacted length; C, compacted length.

The median NC/C ratios with interquartile ranges were calculated for each of the groups. The median maximal NC/C ratio was 1.8 (IQR 1.57, 2.02) for DMD boys with EF > 55% compared to 1.54 (IQR 1.29, 1.78) for the normal group (p < 0.0001). For DMD boys with EF < 55% median maximal NC/C ratio was 2.46 (IQR 1.84, 3.04) compared to 3.69 (IQR 2.81, 4.23) for the LVNC group (p < 0.0001) (Figure 4).

Of the 27 DMD boys who met criteria for LVNC (NC/C ratio > 2.3 in any one segment), the mean number of positive segments was 2.2. This can be compared to the 15 patients with primary LVNC who had a mean of 3.4 positive segments. The median maximal NC/C ratio for all 27 DMD boys that met criteria for LVNC was 3.0 (IQR 2.61, 3.26).

### LGE assessment

The presence of LGE was assessed in 90 of the 96 DMD boys. LGE was positive for 30 of these 90 DMD boys. Of the 30 with LGE, 9 (30%) had normal left ventricular function and 21 (70%) had depressed function. There were 12 of 30 (40%) with NC/C ratios greater than 2.3. Eleven of the 12 subjects with both LGE and LVNC had depressed left ventricular function. The segments that were affected for the 30 with LGE were most commonly segments 5, 6, 11, 12, and 16. The most common segments with a NC/C ratio greater than 2.3 were again 11, 12, and 16. In the 12 patients with LGE and LVNC, there were a total of 30 segments with a NC/C greater than 2.3, 22 or 73% of these segments were also positive for LGE. 12 of the 15 patients with primary LVNC had LGE assessed. There was no evidence of LGE in any of these 12 patients. None of the normal patents had evidence of LGE.

### LVNC in DMD (Analysis of serial studies)

**Figure 5:**
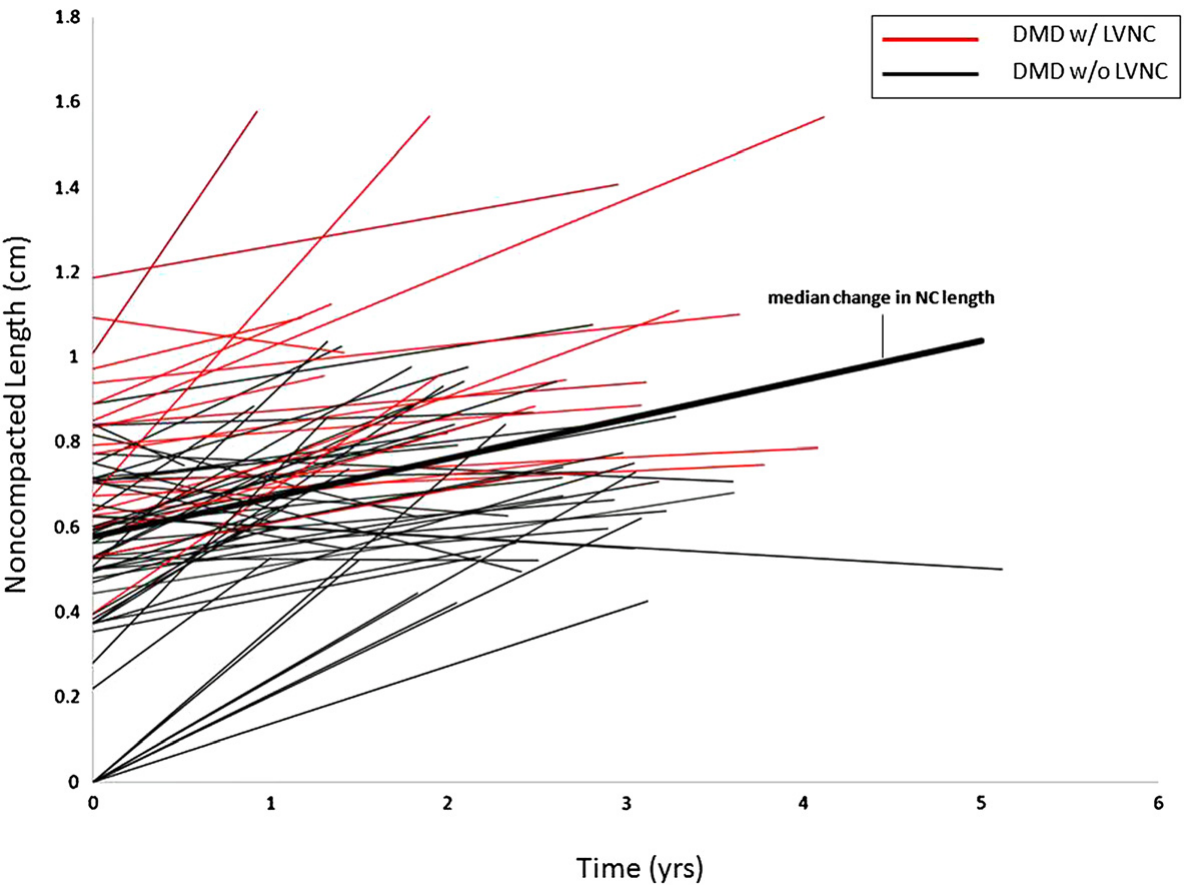
**Noncompacted length vs time.** This graph shows the progression of noncompacted length over time in all DMD patients that longitudinal data was available. There is an increase in noncompacted length over time. DMD, Duchenne Muscular Dystrophy; NC, noncompacted length.

**Figure 6:**
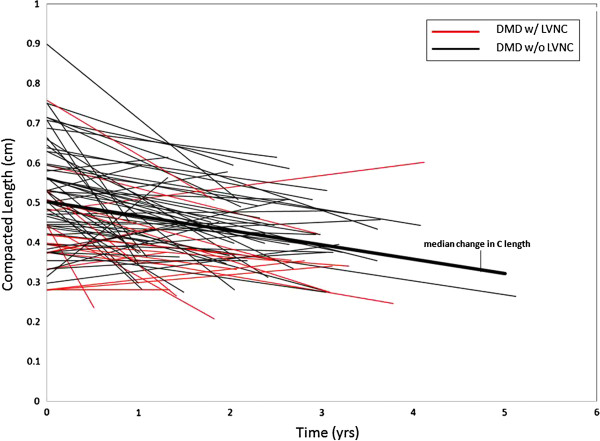
**Compacted length vs time.** This shows the progression of compacted length over time in all DMD patients that longitudinal data was available. There is a decrease in compacted length over time. DMD, Duchenne Muscular Dystrophy; C, compacted length.

**Figure 7:**
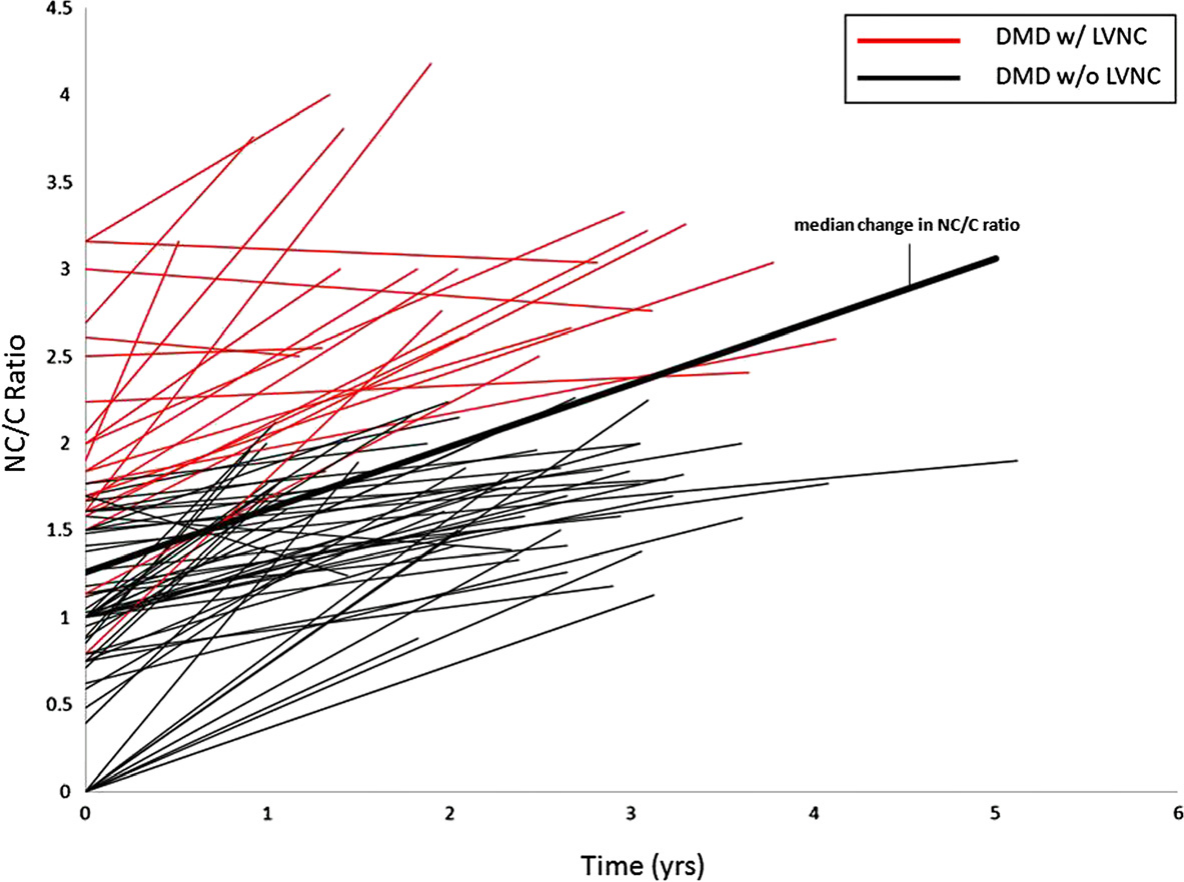
**Noncompacted to compacted ratio vs time.** This graph shows the progression of NC/C ratio over time in all DMD patients that longitudinal data was available. There is an increase in NC/C ratio over time. DMD, Duchenne Muscular Dystrophy; NC, noncompacted; C, compacted length.

There was longitudinal data available for 78 of the DMD boys. The median rate of change in NC length per year was +0.92 mm (IQR 0.34, 2.04). The median rate of change in C length was −0.36 mm (IQR 0.78, 0). The median rate of change in NC/C ratio per year was +0.36 (IQR 0.14, 0.68). In DMD patients with serial data available, the noncompacted layer increased in thickness as the compacted layer decreased in thickness, leading to an increase in NC/C ratio (Figures 5, 6, and 7).

### Pathologic correlation

**Figure 8:**
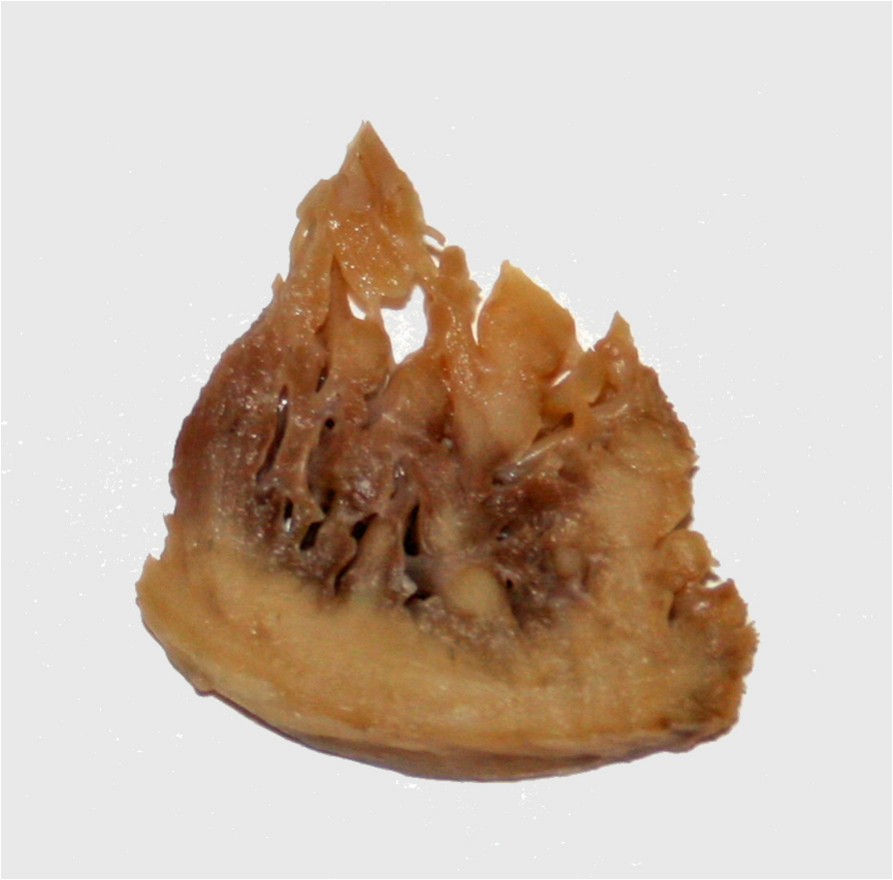
**Pathologic specimen showing LVNC in a DMD patient.** The image shows a piece of myocardium removed from the apex during implantation of a left ventricular assist device. There is extensive noncompacted myocardium extending form the compacted myocardium in finger-like projections. DMD, Duchenne Muscular Dystrophy.

All three patients with available pathology were confirmed to have LVNC by standard pathologic criteria. An example myocardial wedge is shown in Figure 8. There is extensive non-compacted myocardium and relatively thin areas of compacted myocardium. The DMD patient showed extensive areas of fibrosis consistent with typical changes of DMD cardiomyopathy.

### Inter/Intra observer variability

Inter-observer and intra-observer variability was measured on 10% of patients as described above. Intra-observer variability for NC/C ratio had an ICC mean of 0.68 for all segments, which indicates a strong agreement. The inter-observer variability had an ICC mean of 0.97, which indicates a very strong agreement between observers.

## Discussion

This study shows a markedly elevated prevalence of LVNC in a large DMD population with 28% having a NC/C ratio > 2.3 in at least one cardiac segment. DMD boys with normal global function have a much lower prevalence of LVNC than those with decreased systolic function. There is an increase in the non-compacted length with a decrease in compacted length over time in the majority of DMD boys whether or not they met criteria for LVNC.

Several case reports and small case series have found an association of LVNC with neuromuscular disease. This is the first study to describe the prevalence of LVNC within a large DMD population. Stollberger and colleagues described a cohort of 62 patients with LVNC in which 49 had a full neurological work-up. Remarkably, 40 of these patients were found to have some type of myopathy [4]. Finsterer et al. described a case of a patient with DMD who had evidence of LVNC [25].

The phenotypic noncompaction described in this population of DMD is much more prevalent than would be expected in the general population by autopsy or non-invasive imaging. Boyd et al. described deep trabeculations in only 4% of 474 normal hearts on autopsy [26]. Kohli et al. described a cohort of CMRs of those diagnosed with dilated cardiomyopathy and found that ~24% of them met criteria for LVNC [27]. There is sparse data about the prevalence of LVNC diagnosed via echo, and even less in children. In one adult study there was a 3.7% prevalence of definite or probable LVNC found in those with LVEF </= 45% and a 0.26% prevalence for all patients referred for echocardiography over a one year period [28]. Whether this represents an under-recognized population of those with LVNC that then develop poor function and dilation or that it is actually a compensatory mechanism of the failing ventricle has yet to be seen.

Although DMD related heart disease has been historically classified as a dilated cardiomyopathy, it has recently been described as a unique entity characterized by our group as left ventricular functional decline without significant ventricular dilatation until late in disease progression. It was shown that despite normal LVRI (left ventricular remodeling index), myocardial function assessed by CMR strain decreases with age [29]. The LVNC associated with DMD is a progressive phenotype. As DMD associated cardiac disease progresses, LVNC may develop secondary to thinning of the compacted myocardium and resulting in remodeling that appears as LVNC. This apparent increase in trabeculations and thinning of the compacted layer results in an increase in NC/C ratio (Figures 5, 6 and 7). This pattern of increased trabeculation with thinning of the compacted layer seems to occur in most DMD patients over time, further supporting that this is a progressive phenotypic change characteristic of the myocardial degradation.

Advanced DMD associated heart disease has also been described to have thinning and fibrofatty replacement of the apical and mid- LV free wall with markedly positive late gadolinium enhancement (LGE) in these segments on CMR [24,30]. As Figure 2 shows, the segments with NC/C ratios greater than 2.3 are very similar to those segments previously described as being positive for LGE. In our patients, LGE was found in the same segments as the LVNC as described above. One possible explanation is that as the compacted tissue scars and thins, the non-compacted tissue becomes more prominent.

This study is limited by being retrospective. This study is also limited by a lack of universal imaging criteria for the LVNC phenotype. Different criteria for LVNC would yield slightly different prevalence numbers for this population. This group of DMD patients falls in a relatively narrow age range with no patients less than 7 years of age and relatively few older patients.

## Conclusions

Although these data show that the progression of DMD associated heart disease includes the development of LVNC, these patients likely do not have classic primary LVNC cardiomyopathy. Rather, the changes that occur late in the disease process are characterized by marked alteration in myocardial structure secondary to fibrofatty replacement of myocardial cells and/or a compensatory mechanism due to cardiac dysfunction. This data definitively shows DMD patients that originally had normal NC/C ratios later developed trabeculations as the disease progressed. This may give some insight to the high prevalence of noncompacted tissue of those with cardiac dysfunction. It also raises the broader question of whether LVNC in patients with LVNC cardiomyopathy and dilated ventricles is a primary or secondary process.

## Abbreviations

LVNC: Left ventricular non-compaction; LV: Left ventricular; EF: Ejection fraction; DMD: Duchenne muscular dystrophy; CMR: Cardiac magnetic resonance imaging; NC: Non-compacted; C: Compacted; LGE: Late gadolinium enhancement; NC/C: Non-compacted to compacted ratio; IQR: Interquartile range.

## Competing interests

There are no competing interests for any of the authors to disclose.

## Authors’ contributions

CS conceived of the study, and participated in its design and coordination, carried out all data collection and helped to draft the manuscript. MT participated in the design and coordination of the study and helped to draft the manuscript. WM participated in the design and coordination of the study and helped to draft the manuscript. LC helped with study design. EK provided statistical support. JP provided statistical analysis. DWB helped with design of the study and helped to draft the manuscript. KH conceived of the study, and participated in its design and coordination, and helped to draft the manuscript. All authors read and approved the final manuscript.
